# Future Needs in Mast Cell Biology

**DOI:** 10.3390/ijms20184397

**Published:** 2019-09-06

**Authors:** Gilda Varricchi, Amato de Paulis, Gianni Marone, Stephen J. Galli

**Affiliations:** 1 Department of Translational Medical Sciences (DISMET), University of Naples Federico II, 80138 Naples, Italy; 2 Center for Basic and Clinical Immunology Research (CISI), University of Naples Federico II, School of Medicine, 80138 Naples, Italy; 3 WAO Center of Excellence, 80138 Naples, Italy; 4 Institute of Experimental Endocrinology and Oncology “Gaetano Salvatore” (IEOS), National Research Council (CNR), 80138 Naples, Italy; 5 Departments of Pathology and of Microbiology and Immunology, Stanford University School of Medicine, Stanford, CA 94305-5176, USA

**Keywords:** allergy, atherosclerosis, cancer, cancer-related inflammation, mast cell, myocardial infarction, predictive biomarker, tumor-associated mast cells

## Abstract

The pathophysiological roles of mast cells are still not fully understood, over 140 years since their description by Paul Ehrlich in 1878. Initial studies have attempted to identify distinct “subpopulations” of mast cells based on a relatively small number of biochemical characteristics. More recently, “subtypes” of mast cells have been described based on the analysis of transcriptomes of anatomically distinct mouse mast cell populations. Although mast cells can potently alter homeostasis, in certain circumstances, these cells can also contribute to the restoration of homeostasis. Both solid and hematologic tumors are associated with the accumulation of peritumoral and/or intratumoral mast cells, suggesting that these cells can help to promote and/or limit tumorigenesis. We suggest that at least two major subsets of mast cells, MC1 (meaning anti-tumorigenic) and MC2 (meaning pro-tumorigenic), and/or different mast cell mediators derived from otherwise similar cells, could play distinct or even opposite roles in tumorigenesis. Mast cells are also strategically located in the human myocardium, in atherosclerotic plaques, in close proximity to nerves and in the aortic valve. Recent studies have revealed evidence that cardiac mast cells can participate both in physiological and pathological processes in the heart. It seems likely that different subsets of mast cells, like those of cardiac macrophages, can exert distinct, even opposite, effects in different pathophysiological processes in the heart. In this chapter, we have commented on possible future needs of the ongoing efforts to identify the diverse functions of mast cells in health and disease.

## 1. Introduction

The functions of mast cells are still not fully understood, now over 140 years since their description by Paul Ehrlich in 1878 [1]. However, efforts to discern their development have revealed two pathways resulting in mast cells, at least in mice: those which govern the generation of progenitors in the yolk sac and those which modulate the differentiation of precursors in the bone marrow [2,3,4,5]. Similarly, it is now clear that some mast cell populations can undergo relatively rapid changes in population size, notably including so-called “mucosal mast cells” in the mouse gastrointestinal tract [6] or both “mucosal” and “connective tissue” mast cells in animals provided with recombinant stem cell factor [7,8]. By contrast, other mast cells can persist for years, at least in laboratory rodents [9].

Unsurprisingly, a number of studies have attempted to identify distinct “subpopulations” of mast cells. These range from the very old [10,11] to more modern efforts that have documented multiple morphological “types” of mast cells (work extended by Enerback [12]) to those that are based on a relatively small number of biochemical characteristics, such as the mast cell’s content of tryptase vs. chymase [13]. More recently, additional “subtypes” of mast cells have been described, based on the comprehensive analysis of the transcriptome of individual anatomically distinct mouse mast cell populations [14]. Indeed, some time ago, the idea was discussed that: (1) given the extraordinarily long life span of some mast cells, and, (2) in light of their ability to respond with phenotypic alterations in response to so many internal and external signals, it seems possible that mast cells encountered in vivo are ”tunable”, at least for some products or functions [15].

What then are the “key features” which must be taken into account in assigning mast cells to phenotypically different “subtypes”? And how stable are these phenotypes, particularly for mast cells in vivo? These are just some of the questions to address when considering the complexities of discerning distinct functions of mast cells in humans, especially in non-IgE-mediated processes.

In this chapter, we will review particularly the broad range of possible roles of mast cells in human diseases, especially in malignancies or cardiovascular disorders.

## 2. The Mast Cell in Homeostasis

Although mast cells are regarded as cells that are potently able to disturb homeostasis, for example in the case of food-induced anaphylaxis, this is not their only function. Indeed, in certain circumstances, mast cells can contribute to the restoration of homeostasis.

Grimbaldeston et al. [16] showed, using mast cell-deficient WBB6F_1_-*Kit^W/W-v^* mice and C57BL/6-*Kit^W-sh/W-sh^* mice, that mast cells can dampen the extent of either severe contact hypersensitivity (CHS) reactions induced by urushiol (a toxin produced by poison ivy or poison sumac) or severe responses to ultraviolet B irradiation. Furthermore, evidence was provided that some of this mast cell-dependent suppression of the extent of inflammation and tissue damage was due to mast cell production of IL-10. Recently, Reber et al. [17] showed that this “mast cell-dependent” suppression of severe CHS also could be detected when the CHS was elicited by dintrofluorobenzene (DNFB) and when the experiments were done using more “modern” mast cell-deficient mice, namely, *Cpa3-Cre*; *Mcl-1^fl/fl^* mice or *Mcpt5-Cre+* mice. Furthermore, in vivo imaging studies showed that mast cell IL-10 expression was markedly augmented in the mast cells participating in severe, as opposed to mild, CHS reactions to DNFB [17].

The finding that IL-10 production by mast cells can help to dampen inflammation was also reported by Soman Abraham’s group [18]. That study investigated the participation of urinary tract mast cells in bacterial infection of the bladder [18]. The set of signals that inform mast cells that they should upregulate IL-10 production, in either setting, is currently unknown. However, these findings suggest that one function of the mast cell may be to maintain homeostasis of tissues by helping to dampen strong reactions, in part by expressing higher amounts of certain products (e.g., IL-10).

## 3. Protective Roles of Mast Cells

There are two settings in which some mast cell functions appear to be protective. In both cases, the data come from studies of different varieties of mast cell-deficient mice. While early work in these models employed older types of mast cell-deficient mice (i.e., mast cell-deficient WBB6F_1_-*Kit^W/W-v^* mice and C57BL/6-*Kit^W-sh/W-sh^* mice), more recent work with more modern varieties of mast cell-deficient mice has produced similar findings.

First, mast cells have been associated with primary infections to certain parasites, including *Strongyloides* species. Two recent studies, which used different types of c-Kit independent mast cell-deficient mice, have confirmed a role for mast cells in reducing the length of primary infections with *Strongyloides ratti* [19] or *S. brasiliensis* [20]. Notably, little or no role of mast cells was detected in either study during secondary infections with the parasite. These recent studies therefore confirm and extend prior work, employing mast cell-deficient WBB6F_1_-*Kit^W/W-v^* mice, which also suggested a role for mast cells (and IL-3) in limiting the length of infection with *S. brasiliensis* [21].

Second, mast cells have been shown to be important for the full expression of both primary and secondary responses to the venoms of the honeybee and the Russell’s viper [22,23,24]. In the case of primary responses, different mast cell proteases appear to play important roles in mediating resistance to some venoms. For example, carboxypeptidase A appears to play an important role in mediating primary resistance to either the whole venom of the snake, *Atractaspis engaddensis* [22] or to a major toxin in the venom, sarafotoxin [22,25]. By contrast, mouse mast cell protease 4 (thought to be equivalent to human chymase) appears to be important in primary immune responses to the venoms of the Gila monster lizard (*Heloderma suspectum*) and to two species of highly toxic scorpions [26].

Notably, the World Health Organization recently designated envenomation by snake bite to its list of category A neglected tropical diseases [27]. Anderson et al. [28] tested the ability of recombinant human tryptase β to detoxify six different venoms from phylogenetically distinct snakes, using a zebrafish embryo model system. Human mast cell tryptase β, but not other tested proteases (human mast cell chymase or CPA), detoxified each of these six venoms by mass spectrometry, limiting their ability to kill the zebrafish embryos. This result is especially interesting because, unlike other serine proteases, tryptase β is active as a tetramer, thereby preventing it from degrading proteins too large to be accommodated within its central pore. Anderson et al. [28] carefully noted that only the zebrafish embryo toxicity model was used in their study, and that different toxins (possibly with increased resistance to tryptase digestion) might participate in toxicity in humans; that injection of relatively large amounts of recombinant tryptase β might result in adverse effects; and that satisfactory methods had to be developed to store and apply the enzyme in the field. Nevertheless, the work is consistent with the idea that recombinant human tryptase β could potentially be used as a first aid treatment for those bitten by a variety of poisonous snakes.

## 4. Mast Cells in Cancer

Rudolf Virchow first made a connection between inflammation and cancer in the 19th century, based on the observation that tumors often arose at sites of chronic inflammation and that immune cells were present in tumors [29]. The presence of mast cells in human tumors was first reported by Paul Ehrlich [1] and was extended by Eugene Westphal [30]. Tumorigenesis is a process characterized by the accumulation of genetic and epigenetic alterations [31], and the tissue microenvironment plays a central role in maintaining tissue homeostasis or promoting tumors. Thus, the normal microenvironment is considered a barrier to tumorigenesis [32], whereas a collection of “inappropriate” signals (e.g., chemokines, cytokines, reactive oxygen species, reactive nitrogen species, lipid mediators, etc.) can initiate and promote tumors. Indeed, low grade inflammation or smoldering inflammation is a hallmark of cancer [33] and immune cells (macrophages, lymphocytes, neutrophils, mast cells, NK and NKT cells, eosinophils, etc.) are components of the inflammatory microenvironment that can play a role in the development of experimental and human tumors [33,34,35,36].

**Figure 1:**
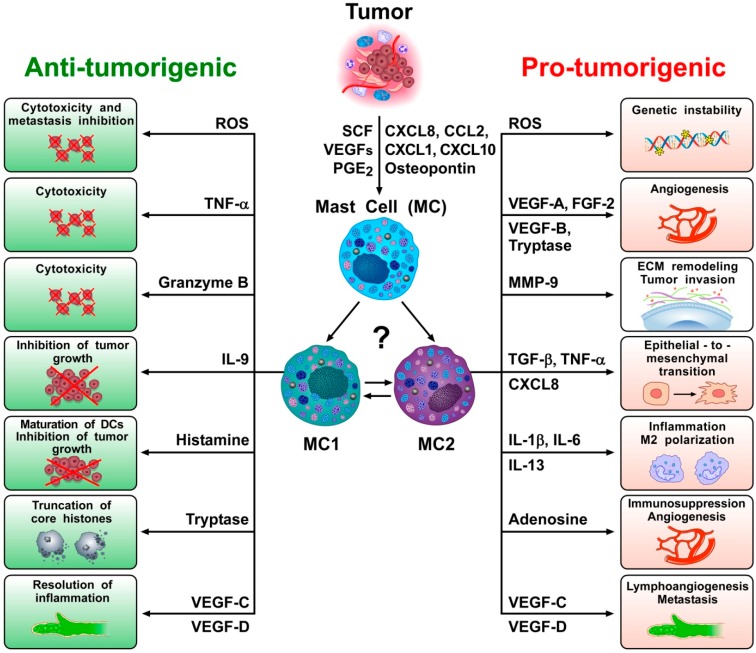
Mast cells can be recruited into tumor microenvironments (TMEs) by several chemotactic molecules (e.g., SCF, VEGFs, PGE_2_, CXCL8, CCL2, CXCL1, CXCL10, osteopontin) produced by tumor or immune cells [49]. However, mast cells in the TME can exert anti-tumorigenic and/or pro-tumorigenic roles. Similarly to neutrophils (N1 and N2) and macrophages (M1 and M2), it is possible that the complex biochemical milieu of the TME (and of tumor cells themselves) can polarize mast cells toward anti-tumorigenic MC1 or pro-tumorigenic MC2 mast cell types. Reactive oxygen species (ROS) are chemically reactive free radicals that potentially function as a double-edged sword [54]. Rodent and human mast cells can produce functionally active ROS [55] and excessive ROS may induce cytotoxic effects that can contribute to tumor regression. Mast cells also can exert direct tumor cytotoxic effects via TNF-α [56,57,58,59] and/or granzyme B [60,61]. IL-9 produced by mast cells can inhibit tumor cell engraftement [62]. Histamine promotes dendritic cell (DC) maturation and inhibits tumor growth [63,64]. Tryptase can be taken up into the nucleus of human melanoma cells causing truncation of histones and inhibition of cell proliferation [65]. Human mast cells also can release lymphangiogenic factors (VEGF-C and VEGF-D) [45,66], and increasing evidence indicates that lymphangiogenesis can play an active role in the resolution of inflammation [67,68]. However, the presence of large amounts of ROS can outstrip the capacity of cellular DNA repair systems, triggering genomic instability and transcription errors that may foster tumor initiation [69]. Mast cells also represent a potentially major source of several angiogenic molecules (VEGF-A, VEGF-B, FGF-2, tryptase) [45,70,71,72,73,74]. In addition, MMP-9 can induce degradation of the extracellular matrix, leading to cancer cell invasion and metastasis [75]. TGF-β, CXCL8 and TNF-α can induce epithelial-to-mesenchymal transition [48,76]. Proinflammatory cytokines such as IL-1β [49,77,78,79,80,81] and IL-6 [82,83] can contribute to chronic inflammation in tumor microenvironment. IL-13 favors M2 polarization of tumor-associated macrophages [77]. Adenosine can be released by activated mast cells and potentiates the release of angiogenic and lymphangiogenic factors from human mast cells [45]. VEGF-C and VEGF-D are the major lymphangiogenic factors produced by human mast cells and can contribute to the formation of metastasis [84,85].

Both solid and hematopoietic tumors are associated with the accumulation of peritumoral and/or intratumoral mast cells [37,38,39,40], suggesting that they play a role in promoting and/or limiting tumorigenesis. Indeed, there is evidence consistent with both of these functions in experimental and clinical studies [37,41]. For example, peritumoral and/or intratumoral mast cell density is increased in different types of human cancer [42]. Moreover, a variety of tumor products can serve to attract mast cells into the tumor microenvironment (TME) (Figure 1), including stem cell factor (SCF) [43,44], vascular endothelial growth factors (VEGFs) [45,46], angiopoietin 1 (ANGPT1) [47], several chemokines (CXCL8, CXCL2, CXCL1, and CXCL10) [46,48,49], prostaglandin E_2_ (PGE_2_) [50,51], TSLP [52], and osteopontin [53].

In several studies, mast cells appeared to play a pro-tumorigenic role in human and experimental tumors [37,86]. Evidence for an anti-tumorigenic role for mast cells was also found in certain tumors [62,87,88], whereas a limited number of studies indicate a non-contributing role of mast cells in certain tumors [37,41,89,90]. As illustrated in Figure 1, these apparently conflicting results are compatible with the hypothesis that different subsets of mast cells and/or different mast cell-derived mediators can play distinct or even opposite roles in tumorigenesis. Future studies should address the possible roles of plasticity/ hypothetical subtypes of mast cells in different cancers, or at different stages of tumorigenesis.

### 4.1. Sublocalization of Mast Cells in Different Stages of Tumor Development

Increasing evidence suggests that the roles of immune cells in tumors vary according to their microlocalization. For instance, sublocalization of lymphocytes can contribute to the prognosis and even to the predictive stratification of patients with breast cancer [91]. Most initial studies evaluating mast cell density in cancer did not examine differences between the sublocalization (the periphery vs. the center) of tumors. Future analyses assessing the spatial distribution of mast cells should evaluate not only their density, but also their state of activation in distinct tumor compartments such as the margin, the tumor core, and macroscopically normal adjacent tissue. It has already been demonstrated that the density of mast cells, as well as several other immune cells, differs in the center of the colorectal carcinoma vs. at the invasive margin [92]. This study also reported differences in mast cell density in different tumor stages. These findings suggest that the microlocalization of mast cells should be investigated in different stages of clinical and experimental tumors.

Indeed, recent results have highlighted marked differences in the phenotypic, genetic and functional characteristics of a variety of tumor-associated immune cells at early vs. late stages of human tumors [93,94,95,96]. There is already some evidence that the role of mast cells may vary according to the stage of tumorigenesis. This has been suggested in studies in melanoma [87], prostate cancer [97,98], non-small cell lung cancer (NSCLC) [99], and colon cancer [100]. However, more studies are needed to characterize the possible metabolic alterations of mast cells residing in different areas, or in different stages, of tumors in order to identify potential therapeutic targets.

### 4.2. Heterogeneity of Mast Cells in Cancer

The potentially dual role (i.e., pro-tumorigenic and anti-tumorigenic) of mast cells in cancer raises the issue of mast cell heterogeneity in cancer. Recently, the marked heterogeneity of several immune cells has been demonstrated by using single-cell sequencing [101,102]. For example, several recent studies demonstrate that the model distinguishing classically polarized anti-tumor M1 and alternatively polarized pro-tumor M2 subtypes [77] incompletely accounts for the in vivo diversity of macrophages [94,102]. Similarly, the predictive potential of the transcriptionally and functionally heterogeneity of intratumoral CD8^+^ T cells has been demonstrated in NSLC [103]. Moreover, two subsets of conventional dendritic cells (DC1 and DC2) [104], neutrophils (N1 and N2) [105,106] and γδ T cells in cancer have been demonstrated [107]. The application of single-cell RNA sequencing (scRNA-seq) platforms will allow high resolution characterization of different subsets of mast cells that accompany tumor initiation and growth. Indeed, one might speculate that at least two major subsets of mast cells, MC1 (meaning anti-tumorigenic) and MC2 (meaning pro-tumorigenic), could play a role in tumorigenesis (Figure 1).

### 4.3. PD-L1 Expression on Mast Cells

The clinical development of checkpoint inhibitor-based immunotherapy has ushered in an exciting area of anti-cancer therapy [108]. Immunotherapy with mAbs targeting cytotoxic T lymphocytes antigen 4 (CTLA-4), or the programmed cell death 1 (PD-1) and its ligand (PD-L1), have achieved impressive success in the treatment of different types of cancer [109]. However, so far, clinical benefit has been achieved in only a minority of patients across many cancer types [110]. Moreover, an immune checkpoint blockade poses a potentially high risk for developing severe immune-related adverse effects [111,112,113,114]. Therefore, identification of truly predictive biomarkers is critical for personalizing patient immunotherapy with immune checkpoint inhibitors (ICIs).

For example, PD-L1 expression on cancer cells as a biomarker for patient selection has been approved for patients who are PD-L1 tumor cell positive [115]. However, multiple studies have not detected a positive correlation between PD-L1 tumor expression and ICI response [109]. Increasing evidence suggests that PD-L1 expression on immune cells (e.g., dendritic cells and macrophages) in TME can determine the efficacy of PD-L1 pathway blockade-mediated tumor regression [116]. Mast cells express PD-L1 and, to a lesser extent, PD-L2 [117,118,119]. Intratumoral mast cells from gastric cancer specimens express PD-1 [120]. Interestingly, PD-L1^+^ mast cells have been found in TME in the early lung adenocarcinoma [119] and the number of PD-L1^+^ mast cells was equal to the density of PD-L1^+^ macrophages. Further studies should evaluate systematically the percentage of PD-L1^+^ and PD-L1^−^ mast cells in the tumor core, tumor margin and in non-malignant distant tissue. The possibility should be considered that the selection of patients for anti-PD-L1/PD-1 therapy could be based on PD-L1^+^ expression on immune cells, including mast cells in TME.

### 4.4. Mast Cells in Tumor Draining Lymph Nodes

Mast cells are rarely found in normal lymph nodes [121,122], but the density of mast cells is markedly increased in metastatic draining lymph nodes [38,123]. This suggests that mast cells and/or their progenitors can migrate to tumor draining lymph nodes (TDLNs), where they might act as non-professional antigen presenting cells (APCs) [121,124,125]. The mechanisms regulating the trafficking of mast cells/mast cell progenitors into TDLNS, and their contributions to the evolving microenvironment of the metastatic niche, remains poorly understood. High-dimensional analysis, particularly single-cell RNA-seq, will be necessary to characterize the mast cell lineage in TDLNs.

### 4.5. Mast Cell Proteases in Cancer

Mast cell secretory granules contain various bioactive mediators, notably including several proteases such as tryptase, chymase, and carboxypeptidase A3 (CPA3) [126]. The contribution of such mast cell proteases to cancer is starting to be delineated. Pejler and collaborators elegantly demonstrated that tryptase also can be found within the nucleus of mast cells and can truncate core histones [127,128]. More recently, the same group presented evidence that human recombinant tryptase can be taken up into the nucleus of human melanoma cells causing truncation of histones [65]. Interestingly, tryptase reduced melanoma cell growth in vitro by blocking proliferation [65]. Experimental studies in genetically-altered mice indicate that the combined actions of chymase, tryptase and CPA3 protect against the formation of lung metastasis of melanoma [129]. Thus, there was higher expression of melanoma-specific genes in lungs of Mcpt4/Mcpt6/CPA3-deficient vs. wild type mice, and mice with a Mcpt4/Mcpt6/Cpa3-deficiency also exhibited reduced numbers of cells expressing CD1d, an MHC class 1-like molecule important for presenting antigen to invariant NKT (iNKT) cells. These findings suggest that the protective role of mast cell-specific proteases in melanoma dissemination involves a CXCL16/CD1d/NKT cell axis. Future studies should investigate, in additional tumor models, whether mast cell proteases also can have protective roles in the formation of metastasis.

### 4.6. Influence of Microbiota on Mast Cells in Cancer

It is now clear that the microbiota can modulate the innate [130] and the adaptive immune system [131]. Mast cells are located strategically at host-microbiota interfaces, including the skin and the mucosae of the lung and of gastrointestinal tract. Mast cells have the ability to sense microorganism or their metabolic products and to translate the signals into host pathophysiological responses [132]. Moreover, microbiota-derived metabolites signal to distant organs, which enables the host microbiota to alter the immune system of the host [133].

The microbiota is also involved in the initiation, progression and dissemination of cancer both at epithelial barriers and in sterile tissues [134,135]. Recent evidence indicates that lung cancer development is associated with local dysbiosis and chronic inflammation with neutrophil infiltration and tumor cell proliferation [136]. Importantly, the intestinal microbiota has a major influence on the effectiveness of anticancer immunosurveillance and thereby contributes to the therapeutic activity of ICIs [137,138,139,140]. The skin microbiota can promote mast cell maturation by triggering SCF production by keratinocytes [141]. Mast cells also can be activated by a variety of bacterial and viral products to release a wide spectrum of proinflammatory and immunoregulatory mediators [66,142,143,144]. Further studies will be needed to fully investigate the potentially diverse effects of the microbiota on mast cell functions in tumorigenesis.

### 4.7. Adipose Tissue Mast Cells and Cancer

Up to 49% of certain types of cancer are now associated with obesity [145], and weight loss can reverse cancer risk [146]. Obesity-associated inflammation can dramatically alter immune cell composition in different tissues [147,148]. Indeed, there is evidence that obesity leads to metabolic reprogramming of immune cells and limits anti-tumor responses [149]. White adipose tissue (WAT) from humans and mice contains mature adipocytes and several immune cells including mast cells [150,151]. WAT also is a reservoir of functional mast cell progenitors [152,153,154]. Moreover, TNF-α of mast cells from adipose tissue can induce apoptosis of cancer cells in vitro [56]. It therefore would be interesting to evaluate whether obesity can influence mast cell responses during cancer initiation and progression.

### 4.8. C5a Receptor Signaling in Mast Cells and Cancer

Complement is a critical component of humoral immunity implicated in recognition and elimination of pathogens and damaged cells, opsonization, and anaphylactoid reactions [155]. Complement is also a critical component of humoral immunity involved in cancer development [156,157]. Human mast cells express two C5a receptors (C5aR1 and C5aR2) [158] whose activation induces the release of pro-inflammatory mediators [158,159,160,161]. C5aR1 signaling in mast cells and macrophages is thought to foster carcinogenesis and C5aR1 inhibition improves response to chemotherapy [162]. Moreover, blocking C5aR1 signaling promotes the anti-tumor efficacy of PD-1/PD-L1 blockade [163]. These results provide evidence that C5a and signaling pathways downstream of the C5aR can regulate carcinogenesis by promoting inflammation. Accordingly, the role of C5aR1-expressing mast cells as promoters of cancer-associated inflammation merits further study.

### 4.9. IL-1, IL-33 and Mast Cells in Cancer

Inflammasome activation and IL-1β accelerate tumor invasiveness, growth, and metastatic spread [78]. In IL-1β^−/−^ mice, neither local tumors nor lung metastases develop after localized or intravenous inoculations of melanoma cell lines, suggesting that this cytokine participates in tumorigenesis in such models [79]. The Canakinumab Antiinflammatory Thrombosis Outcomes Study (CANTOS) evaluated the efficacy of a human mAb targeting IL-1β in patients who had myocardial infarction [164]. Additional data from the CANTOS trial suggested that inhibition of IL-1β with increasing doses of canakinumab was associated with a dose-dependent reduction of lung cancers [165]. In a prior study, the IL-1 receptor antagonist anakinra reduced the progression of smoldering myeloma [166]. IL-1 can induce release of IL-6 from human mast cells [80] and of IL-8 [81] contributing to angiogenesis [167]. Moreover, evidence from mice indicates that the mast cell-derived IL-1β can mediate malignant pleural effusion [49]. Additional studies are needed to elucidate the role of IL-1/mast cell interactions during cancer development, and to clarify whether the IL-1 blockade may represent a valid approach in different types of cancer.

IL-33 is in the IL-1 family of cytokines that exerts pleiotropic activities [168]. In response to tissue damage, infection or necrosis, IL-33 is released in the extracellular space where it functions as an alarmin for the immune system [36]. IL-33 activates its receptor complex (ST2: IL-1RAcP) on a variety of immune cells including human mast cells [169] and basophils [170]. Moreover, IL-33 synergizes with IgE- and non-IgE-dependent stimuli to release cytokines [171] and VEGF from human mast cells [70]. It has been reported that IL-33 can upregulate the Fcγ receptor type IIa and can synergistically enhance immune complex-triggered activation of human mast cells in vitro [172]. Single cell analysis demonstrated that IL-33 could increase both the number of degranulating and chemokine-producing mast cells and the magnitude of individual mast cell response [173].

Increasing evidence indicates that the IL-33/ST2 axis plays a role in tumor immunity [36]. However, both pro-tumoral and anti-tumoral functions have been reported, and IL-33 may differently influence tumor immunity depending on the tumor type, relevant immune cell targets, and microenvironmental factors [174].

Mast cell activation by IL-33 [169,175] may occur in a variety of tumor types. In mouse skin cancers, mast cells accumulated near IL-33-expressing fibroblasts in UV-exposed skin [176]. In the ApcMin/+ mouse model, an IL-33 deficiency reduced tumor burden [177] and decreased mast cell density in the colonic polyps, as well as suppressed the gene expression of mast cell proteases and cytokines that promote angiogenesis, Treg function, and MDSC recruitment within the TME [178,179,180].

IL-33 also can exert anti-tumoral and anti-metastatic effects by conditioning other immune cells. For example, IL-33 appears to inhibit B16 F10 melanoma through recruitment and activation of eosinophils [181]. Although the role of IL-33 in tumorigenesis and cancer immunity thus remains controversial, further studies should help to elucidate whether IL-33/ST2 signaling in mast cells can contribute to human or experimental tumorigenesis.

### 4.10. Acidity, Hypoxia and Potassium as Modulators of Mast Cell Response and Tumor Progression

Typically, studies of immune cells are performed on cells isolated from peripheral blood or tissues, where nutrients and O_2_ are generally abundant and pH is neutral. However, key features of the TME are low pH and the accumulation of lactate (a byproduct of the anaerobic glycolytic pathway), localized hypoxia [182,183] and elevated potassium [184,185]. These factors can alter the cellular responses of tumor-associated immune cells including macrophages [186,187], T cells [184,185] and mast cells [188]. For example, oxygen-glucose deprivation can induce mast cell degranulation and the release of histamine [189] and of IL-6 [190] and lactic acid can inhibit IL-33- and lipopolysaccharide (LPS)-mediated cytokine and chemokine production from mouse and human mast cells [188,191]. By contrast, another study reported that an acidic environment augments IgE-mediated production of IL-6 and IL-13 from mouse mast cells [192]. Additional studies clearly are required to examine the effects of lactic acid, hypoxia, and extracellular potassium on the release of proinflammatory and angiogenic mediators from different types of mast cells activated by tumor-associated stimuli.

### 4.11. Canonical and Non-Canonical Angiogenic and Lymphangiogenic Factors from Mast Cells

Angiogenesis is a hallmark of cancer and is indispensable for fostering tumor growth [193]. In addition to tumors, several immune cells produce angiogenic factors within the TME [167]. Tumor lymphangiogenesis occurs both in the primary tumors and/or in the formation of metastasis [84,194]. VEGF-A is the most potent canonical pro-angiogenic molecules, while VEGF-C and -D are the canonical lymphangiogenic factors [195,196]. Mast cells represent a potential source of VEGF-A [45,70,71,72,73,74] and only tissue resident immune cells such as macrophages [197] and mast cells, are thought to produce lymphangiogenic factors [45]. Mast cells also express two isoforms of VEGF-B [45] which can play roles in tumorigenesis [100]. Mast cells also can exert a pro-tumorigenic role through the production of CXCL8 [48] and MMP-9 [75].

There is also evidence that cysteinyl leukotrienes (cys-LTs), major lipid mediators of mast cells, can promote tumor angiogenesis via the activation of the cys-LT receptor, CysLT_2_R [198,199,200]. Interestingly, a CysLT_2_R antagonist reduced tumor growth in mice, suggesting that this receptor could be a possible target in the modulation of tumorigenesis [200].

Although lymphangiogenesis has been canonically associated with the formation of metastasis, there is increasing evidence that the lymphatic system also can contribute to the resolution of inflammation [67,68]. In addition, there is evidence that lymphatic vessels can exert an immunomodulatory role and participate in immune surveillance [68]. More investigation is necessary to verify the possibility that the production of lymphangiogenic factors from tumor-associated mast cells can contribute not only to the formation of metastasis, but, in certain conditions, to the resolution of tumor-associated inflammation.

## 5. Mast Cells in Cardiovascular Diseases

**Figure 2:**
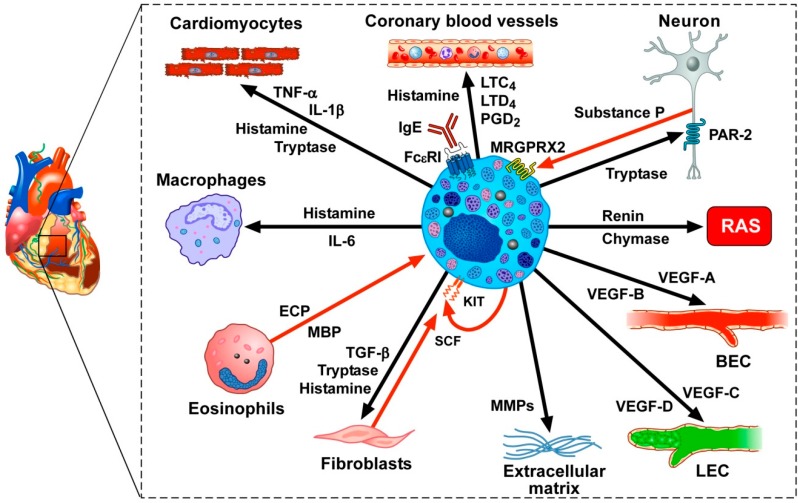
In the human heart, mast cells are located in the myocardium, in atherosclerotic plaques, in close proximity to nerves and in the aortic valve. The density of myocardial mast cells is increased following myocardial infarction [219] and in patients with cardiomyopathies [204]. Several vasoactive mediators that can be released by activated cardiac mast cells (i.e., histamine, PGD_2_, LTC_4_, LTD_4_) exert profound hemodynamic effects on human coronary blood vessels [220,221,222]. Cardiac mast cells often are in close proximity to sensory nerve fibers. Tryptase can activate the PAR-2 receptor on sensory nerve fibers which can release the neuropeptide substance P [223], which, in turn, can activate the adventitial mast cells [206], presumably through the engagement of the Mas-related G-protein-coupled receptor member X2 (MRGPRX2). Human cardiac mast cells contain and release renin and chymase that cleave angiotensinogen and angiotensin I (ANG I), respectively to form ANG II, thus potentially participating in the homeostatic control of the cardiac renin-angiotensin system (RAS) [224,225]. Immunologically-activated human cardiac mast cells release angiogenic (VEGF-A and VEGF-B) and lymphangiogenic factors (VEGF-C) [66,226,227], which can act on blood endothelial cells (BECs) and lymphatic endothelial cells (LECs), respectively. Matrix metalloproteinases (MMPs) released from mast cells can contribute to extracellular matrix remodeling leading to cardiac fibrosis [228]. Tryptase, TGF-β, and histamine activate fibroblasts to produce collagen [228,229]. Fibroblasts also produce stem cell factor (SCF), the main growth and differentiating factor for mast cells that acts via the engagement of the KIT receptor [8]. Human myocardial mast cells contain and release SCF, which might represent an autocrine factor sustaining mast cell hyperplasia in cardiac disorders [204]. Eosinophils and eosinophil granule proteins (i.e., eosinophil cationic protein (ECP) and major basic protein (MBP)) have been detected in endomyocardial biopsies from patients with hypereosinophilia [230,231]. ECP and MBP can induce the release of proinflammatory mediators from human cardiac mast cells [159]. Human cardiac mast cells interact with macrophages through the release of histamine and IL-6 (Marone, unpublished results). Mast cell tryptase modulates cardiomyocyte contractility through the engagement of the PAR-2 receptor [219]. Histamine, tryptase, TNF-α, and IL-1β can modulate several functions of cardiomyocytes [219,232]. Finally, heterogeneous and ontogenetically diverse macrophages reside in the healthy heart, and accumulate in increased numbers in diseased hearts [233].

Recently, there has been increased interest in the potential involvement of mast cells in cardiometabolic disorders [201,202,203]. In the human myocardium, mast cells are located in close proximity to cardiomyocytes, coronary microvessels, nerves, and lymphatic vessels [159,204,205,206,207]. Figure 2 illustrates some of the mediators produced or acting on cardiac mast cells. Unsurprisingly, mast cells and their powerful vasoactive and proinflammatory mediators (e.g., histamine, tryptase, chymase, prostaglandin D_2_ (PGD_2_) and cysteinyl leukotriene C_4_ (LTC_4_)) were considered to be net detrimental in atherosclerosis [151,207,208,209,210], myocardial infarction [211,212], myocarditis [213,214,215], dilated cardiomyopaties [159,201], hypertension [216,217] and thrombosis [218].

However, recent studies have revealed that cardiac mast cells may participate both in physiological and pathological processes in the heart. There is evidence that mast cells can participate in the formation of new blood vessels (angiogenesis) as well as lymphatic vessels (lymphangiogenesis), which are vital processes during the embryologic development of the heart and during myocardial wound healing after myocardial infarction [234,235]. Moreover, immunologic and superantigenic stimuli can induce the release of proangiogenic (VEGF-A) and lymphangiogenic (VEGF-C) factors from primary human cardiac mast cells [66,226]. Notably, the production of VEGF-C by human cardiac mast cells can exert a potentially cardioprotective effect, since activation of the cardiac lymphatics improves the resolution of inflammation [67,236] and has an essential role in counteracting myocardial edema [68,237,238]. Interestingly, the activation of the protease activated receptor 2 (PAR-2) on cardiomyocytes by tryptase following experimental myocardial infarction also can exert a net protective effect [219].

Moreover, cardiac mast cells are located near neuropeptide substance P-positive sensory nerves both in the myocardium and in the adventitial layers of the coronary arteries [206,239]. Substance P, released by sensory nerves, can activate adventitial mast cells [206], presumably, through the engagement of MAS-related G protein-coupled receptor-X2 (MRGPRX2) receptor to release several proinflammatory mediators. In the mouse, a similar system has also been identified as an underlying mechanism that promotes the development of type 2 skin inflammation in response to certain allergens, including house dust mites and *Staphylococcus aureus* [240].

Mast cells also can induce a protective response against microbial pathogens and other noxious substances by releasing soluble mediators (e.g., TNF-α, proteases) [126,241,242]. Indeed, MRGPR-mediated activation of local mast cells has been shown to help clear cutaneous bacterial infection and to protect against reinfection in mice [243]. In humans with chronic rhinosinusitis with nasal polyps, mast cells may help in defense against *Staphylococcus aureus (S. aureus)*, particularly in the presence of *S. aureus* enterotoxin B (SEB), by ejecting extracellular DNA traps (Mast Cell Extracellular Traps: MCETs) [244]. MCETs can also be generated during coronary atherothrombosis in patients who died from myocardial infarction [245]. However, since activated mast cell-formed MCTEs are involved in the elimination of pathogens [246,247], cardiac mast cells also might contribute to the resolution of infectious myocarditis. Additional knowledge regarding the role of MCETs during cardiovascular diseases may provide new strategies for the treatment of these disorders.

In patients with cardiomyopathies, mast cell density in the myocardium is markedly increased [204]. Moreover, the release of preformed and de novo synthesized mediators from cardiac mast cells derived from these patients was increased when compared to results from mast cells of control subjects without cardiovascular disease [204]. Mast cells contain and release several cytotoxic mediators such as TNF-α [56,57,58,59,248] and granzyme B [60,61]. Given the proximity of mast cells to cardiomyocytes and the ability of mast cells to release cytotoxic mediators, there is a possibility that mast cells can contribute to myocardial damage by this mechanism.

However, it is likely that different subsets of mast cells, like those of cardiac macrophages [249,250,251,252], can exert distinct, and even opposite, effects in different pathophysiological process in the heart.

Recent fate mapping experiments demonstrate that mast cells form a highly heterogeneous population of immune cells [3,4], similar to T cells [101,103] and macrophages [94,102]. We speculate that single-cell analyses of different sections of heart will likely identify subsets of myocardial mast cells, and will help to elucidate their functions in different pathophysiological conditions. Indeed, mast cells likely comprise distinct subsets of cells, like subpopulations of resident cardiac macrophages that can promote both injury and repair after myocardial infarction [253].

Accordingly, we think that two questions need to be addressed. Can mast cell precursors, like for T cells [101,103] and macrophages [249,250,251,252], generate functionally distinct subsets of cardiac mast cells with beneficial or harmful activities? Alternatively, do individual differentiated cardiac mast cells have enough plasticity to develop distinct, sometimes opposite, characteristics in response to local and/or systemic environmental stimuli (Figure 2)? Whatever the answer to these questions, the characterization of subpopulations of cardiac mast cells by single-cell RNA-seq, together with analysis of the encoded proteins, may be of importance in efforts to modulate pharmacologically or genetically the injury- or repair-inducing abilities of these cells.

## 6. Outstanding Questions and Conclusions

We have briefly reviewed some of the many potential roles of mast cells in health and disease, with a particular focus on tumor biology and cardiovascular disorders. However, we hope that we also have illustrated how difficult it can be to prove that mast cells have particular roles in vivo. Inbred mice are quite useful for this purpose, in that there now are multiple strains with profound constitutive or inducible mast cell deficiencies—but none of these produce mice that are solely and completely devoid of all mast cells. For example, in some cases, the genetic effects influence basophil numbers and functions, as well as numbers of mast cells [254] and in other cases particular subtypes of mast cells are deleted, without influencing other mast cell populations [255]. In addition, the ablation of mast cells, while in some cases profound, is rarely 100%. Finally, “mast cell-deficient” mice have been derived primarily on the C57BL/6 background and, to a lesser extent, on BALB/c. Accordingly, work with such animals produces results that might be mainly restricted to those strains of inbred mice. The relevance of the findings to the roles of mast cells in other strains of mice, and, more importantly, to humans, remains to be established.

Finally, to our knowledge, no drug has yet been reported that solely and selectively targets mast cells. Therefore, while we have reviewed much work suggesting that mast cells may have significant effects on diverse biological responses in mice or in humans, *proof* of such roles, especially in humans, remains elusive.

## Abbreviations

APC	antigen presenting cell
cys-LT	cysteinyl leukotriene
CTLA-4	cytotoxic T lymphocytes antigen 4
DC	dendritic cells
ECM	extracellular matrix
ECP	eosinophil cationic-protein
FcεRI	high affinity receptor for IgE
ICI	immune checkpoint inhibitor
IL	interleukin
LPS	lipopolysaccharide
mAb	monoclonal antibody
MBP	major basic protein
MDCS	myeloid-derived suppressor cell
MMP	matrix metalloproteinase
NK	natural killer cells
NKT	natural killer T cells
NSCLC	non-small-cell lung cancer
PAR	protease-activated receptor
PD-1	programmed death-1
PD-L1	programmed death ligand 1
PD-L2	programmed death ligand 2
PGD_2_	prostaglandin D_2_
PGE	prostaglandin E
PlGF	placental growth factor
RAS	renin-angiotensin system
SCF	stem cell factor
sc RNA-seq	single-cell RNA sequencing
TDLN	tumor draining lymph node
TGFβ	transforming growth factor β
TME	tumor microenvironment
TNF	tumor necrosis factor
TSLP	thymic stromal lymphopoietin
VEGF	vascular endothelial growth factor
VEGFR	vascular endothelial growth factor receptor
